# Adaptation and validation of the modified weight bias internalization scale (WBIS-M) in Brazilian adults

**DOI:** 10.1371/journal.pone.0328176

**Published:** 2025-07-31

**Authors:** Paula Victoria Sozza, Eva Penelo, Maria Fernanda Laus, David Sánchez-Carracedo, Sebastião Sousa Almeida, Telma Maria Braga Costa

**Affiliations:** 1 Departamento de Psicologia, Universidade de São Paulo, Ribeirão Preto, Brazil; 2 Departament de Psicologia Clínica i de la Salut, Universitat Autònoma de Barcelona, Bellaterra, Barcelona, Spain; 3 Departament de Psicobiologia i de Metodologia de les Ciències de la Salut, Universitat Autònoma de Barcelona, Bellaterra, Barcelona, Spain; 4 Departamento de Ciências da Saúde, Universidade de São Paulo, Ribeirão Preto, Brazil; 5 Curso de Nutrição, Universidade de Ribeirão Preto, Ribeirão Preto, Brazil; Federal University of Ceara, BRAZIL

## Abstract

Internalized weight stigma refers to individuals’ self-stigmatization, leading to self-devaluation. This research aimed to conduct an adaptation and validation study of the Modified Weight Bias Internalization Scale (WBIS-M) for Brazil. A sample of 418 adults (253 women; mean age = 30.8 years, *SD *= 10.4) completed the WBIS-M and measures of anti-fat attitudes, body image, disordered eating, binge eating, and self-esteem. Exploratory factor analyses (EFA) and confirmatory factor analyses (CFA) were conducted to examine the factor structure of the WBIS-M. Measurement invariance across gender was tested with multigroup CFA. Internal consistency was assessed using omega and alpha. Relationships between the WBIS-M scores and external measures were analyzed using Pearson’s and Spearman’s correlations, t-tests, and Cohen’s *d* for effect size. EFA and CFA showed that the 10-item and 1-factor WBIS-M model fit reasonably well (CFI and TLI ≥ .98, SRMR ≤ .06, although RMSEA ≤ .12). Full metric and full scalar invariance evidenced equivalence across genders. The internal consistency reliability coefficients were satisfactory (α and ω = .94). A higher WBIS-M score was linked to greater body dissatisfaction, restrictive/compensatory behaviors, food/weight concerns, binge eating severity, and lower self-esteem. Women and those with a higher BMI had higher WBIS-M scores. Finally, the Brazilian WBIS-M’s 10-item score is a valid and reliable measure for assessing weight self-stigma in adults.

## Introduction

Weight stigma is the social devaluation and defamation of people living with obesity (PLWO), leading to negative attitudes, stereotypes, discrimination, and prejudice [1]. There are conceptual differences between weight stigma (public stigma: e.g., anti-fat attitudes) and internalized weight stigma (self-stigma), as the former reflects an interpersonal concept and the latter an intrapersonal concept. People often readily recall public stigma when thinking about stigma. Negative attitudes, discrimination, and prejudice toward a group represent it. In contrast, self-stigma involves individuals devaluing themselves, damaging their self-esteem, and experiencing mental health issues due to internalizing and accepting negative stereotypes and applying these stereotypes to themselves [2–5].

Internalized weight stigma harms physical, psychosocial, and behavioral health [6]. It is linked to increased inflammatory and stress markers [7], weight gain, and reduced quality of life [6,7]. Psychosocially, it is associated with emotional dysregulation, depression, anxiety, body dissatisfaction, and lower self-esteem [6,8]. Behaviorally, it relates to disordered eating, binge eating, avoidance of healthcare and physical activity [6–9], and increased use of alcohol or other circumstances [9].

Anti-fat attitudes and the internalization of weight stigma are two separate constructs and are not directly associated. The former is explained by attributions made about the “other”, while the latter relates to the “self” [10]. For this reason, studies have shown that internalized weight stigma has weak or insignificant associations with anti-fat attitudes but moderate to strong associations with drive for thinness, binge eating, lower self-esteem, and body dissatisfaction [10–12]. Personal and social experiences contribute to this internalization. For example, the higher weight bias internalization was associated with the belief that thinner body types are more attractive, alongside personal exposure, factors such as lower daily exposure to larger body sizes, having thinner close friends and normalization beliefs suggesting that thinner body types are healthier and more attractive [5].

Although internalization occurs across all weight statuses, young adults [5,7,13,14], women, and those with higher Body Mass Index (BMI) [13–15] show greater internalization. Women often face more pressure and stigmatizing situations related to body weight [5,15].

There are two instruments for assessing weight self-stigma in PLWO. The “Weight Self-Stigma Questionnaire” examines fear of stigma and self-devaluation in PLWO and overweight people [16]. The “Weight Bias Internalization Scale (WBIS)” is an instrument proposed to assess the participants’ beliefs about negative stereotypes and negative self-statements about experiencing overweight and obesity [10].

A modified version, WBIS-M, replaces “overweight” with “my weight” in six of its original 11 items to apply to all weight statuses (e.g., “I am less attractive than most other people because of my weight”). This WBIS-M has shown a unidimensional structure, factor loadings at or above.50, and excellent internal consistency reliability (α = .94) [12]. Several versions of the WBIS-M have been adapted for adults in different countries, such as the 11-item validated version for adults in Greece [17], Spain [18], Turkey [19], the 3-item version in Germany [20] and Arabia [21], and the 10-item version in the U.S. [22].

Concerning the WBIS-M validation studies that maintained all 11 items, a satisfactory unidimensional solution has been reported for the Greek version (factor loadings ranged from.46−.76) [17], for the Spanish version (factor loadings between.44−.91) [18], and the Turkish version (item-total score correlation coefficients between.45−.81) [19]. In addition, excellent internal consistency reliability was found in the studies above (α or ω between.92 and.93). Notably, in all these studies, including the original research by Pearl and Puhl [12], item 1 showed the lowest factor loading.

As for the instruments with fewer items, a 3-item short version of the WBIS-M was proposed for the German population (correlation coefficients with the rest of the items between.80−.87) with an excellent internal consistency reliability for the total score (α = .93) [20], and for the Arab world population, with factor loadings ranging from.78−.90 and an excellent internal consistency (ω = .87; α = .87) [21]. The 10-item version of the WBIS-M for U.S. citizens, with the exclusion of item 1, was validated in two adult groups, and both showed factor loadings above.40 (ranging from.43−.85 and from.53−.88) and a high internal consistency reliability coefficient (α = .93 and.96) [22].

The need to expand weight stigma research globally, particularly in low- and middle-income countries and in different languages, has been highlighted as a critical step in identifying effective strategies to reduce weight stigma [23]. Currently, there are no instruments for assessing internalized weight bias in the Brazilian population across different weight statuses. The only available tool is an adaptation of the original WBIS, translated and semantically validated in a sample of 54 PLWO [24]. There are no quantitative studies on internalized weight stigma in Brazil; only two qualitative studies with women reporting negative feelings, such as body dissatisfaction and guilt for not losing weight [25,26].

Thus, this study was designed to cover these gaps, and the specific aims were: [1] to carry out a cross-cultural adaptation of the instrument from English to Brazilian Portuguese; [2] to provide evidence on content validity by assessing the understanding of the questionnaire through an interview with a group of experts on the subject and through a focus group with Brazilian adults; [3] to evaluate the internal structure of our WBIS-M version in terms of dimensionality using both exploratory and confirmatory factor analysis and measurement invariance across gender of the selected model; [4] to study the internal consistency reliability of derived scores; [5] and to provide evidence on convergent validity through correlations with similar measures as the original version [10,12], and on relation with external variables such as gender, age, and BMI.

Based on previous validation studies, we hypothesize that the WBIS-M will replicate the original 1-factor model and show high positive correlations with body dissatisfaction, moderate and negative correlations with self-esteem, food and weight concerns, restrictive and compensatory practices and binge eating, and low correlations with anti-fat attitudes. We also expect higher internalized weight stigma scores in women than men and at higher BMI and younger age.

## Methods

### 1. Participants and procedure

Initially, 458 individuals aged 18–68 agreed to participate in the study via online recruitment. We excluded data from three participants aged below 18 years old. Additionally, 37 participants who had only answered the sociodemographic questionnaire were also excluded. Therefore, the final sample comprised 418 individuals (Table 1).

**Table 1:** Demographic Characteristics of the Final Sample (*N* = 418).

*Characteristics*	*N*	*Minimum*	*Maximum*	*M*	*SD*
Age (years)	418	18	68	29.9	11.4
BMI (kg/m²)^(a)^	398	15.0	60.2	25.9	5.4
	***n***	***%***			
Gender^(a)^					
Women	253	60.8			
Men	160	38.5			
Other	3	0.7			
Race/ethnicity^(a, b)^					
White	322	77.4			
Brown	65	15.6			
Black	21	5.0			
Yellow	7	1.8			
Indigenous	1	0.2			
Marital status^(a)^					
Single	252	60.9			
Married	98	23.7			
Stable relationship	48	11.6			
Divorced	13	3,1			
Separated	3	0.7			
Educational attainment^(a)^					
Postgraduate	167	40.0			
College/University complete	70	16.8			
College/University incomplete	132	31.7			
High school complete	44	10.6			
High school incomplete	3	0.7			
Elementary school	1	0.2			
Monthly income^(a, c)^					
One to three minimum wages	195	47.1			
Four to five minimum wages	67	16.2			
More than five minimum wages	107	25.8			
Less than one minimum wage	45	10.9			

Note: ^a)^Not all survey participants answered this question;

^b)^The race/ethnicity categories were by the official Brazilian census;

^c)^The value of the minimum wage was R$1,212.

This study followed the guidelines established in the Declaration of Helsinki [27], and ethics approval was obtained from the Institutional Review Board of the last author’s university institution (IRB: 51927421.1.0000.5498).

Invitations were sent to potential participants to complete a survey advertised as “Beliefs about obesity - Validation of the Modified Weight Bias Internalization Scale (WBIS-M)” between June 20 and August 15, 2022. Participants were recruited via email, and advertisements were placed on social media. The invitations to participate in the research were disseminated by various educational institutions, such as universities and graduate programs, which promoted it to their students and staff. Additionally, the researchers shared the research on social media. Information about the project was given to potential participants, and those who agreed to participate were provided with a digital informed consent form, subsequently completing the survey online. The survey was hosted on the REDCap platform. The survey was anonymous, data were treated confidentially and complied with the reference data protection law. All participants took part voluntarily and were not remunerated for participation.

## 2. Measures

### 2.1. Demographics

All participants were asked to report their age, race/ethnicity by official categories in the Brazilian census [28], educational level, income, marital status, and height and weight, which were used to compute BMI as kg/m^2^.

### 2.2. Weight bias internalization

The Modified Weight Bias Internalization Scale (WBIS-M) is an 11-item instrument [12] that assesses the participants’ beliefs regarding negative stereotypes and negative self-statements about the weight that they apply to themselves. Items are rated on a 7-point Likert scale ranging from *“Strongly disagree”* to *“Strongly agree*.” The total score is based on the average of the item responses, after reversal when necessary, ranging from 1 to 7, with higher scores indicating higher internalized weight bias. The psychometric properties in the current sample are an aim of the paper and are therefore reported in the results section.

### 2.3. Anti-fat attitudes

The Brazilian version [29] of the “Social/Character Disparagement” subscale of the Anti-fat Attitudes Test (AFAT) [30] was applied, which has 15 items ascribing socially undesirable personality characteristics to and social disregard for fat people (e.g., If fat people are not hired for a job, the fault is theirs). It uses a Likert-type scale with five options ranging from *“Strongly disagree = 1”* to *“Strongly agree = 5”.* The score is calculated through the average of item responses, and the higher the score, the greater the anti-fat attitude. In the present sample, the 15-item and 1-factor model with CFA showed excellent fit [CFI = .968, TLI = .963, RMSEA = .048 (90% CI:.038−.059), SRMR = .063, χ^2^_(90)_ = 175.8], with standardized factor loadings ranging from.40 to.86; internal consistency reliability was acceptable (Cronbach’s α = .77, mean inter-item correlation.24 and McDonald’s ω = .83).

### 2.4. Body image and disordered eating behaviors

#### 2.4.1. Body dissatisfaction.

To assess concerns over body shape and body image, we used the Body Shape Questionnaire [31], specifically a shortened version (8 items) of the Body Shape Questionnaire (BSQ-8) [32] that had been validated for Brazilian adults. It uses a 6-point response format ranging from *“Never” *= 1 to *“Always” *= 6 (e.g., “Have you ever felt ashamed of your body?”). The score is obtained through the average of item responses; higher values indicate more body dissatisfaction. Nearly excellent fit for the 8-item and 1-factor model with CFA [CFI = .988, TLI = .983, RMSEA = .087 (90% CI:.068−.107), SRMR = .023, χ^2^_(20)_ = 80.4], with standardized factor loadings ranging from.66 to.86; good internal consistency reliability was obtained in the present sample (Cronbach’s α = .90 and McDonald’s ω = .91).

#### 2.4.2. Binge-eating.

Binge eating was measured with the replication of what was proposed by Andrés et al. [11], with four questions based on the DSM-V criteria [33] to assess the presence of binge eating in the last three months (*yes/no*), with or without loss of control (*yes/no*), frequency of binge eating with loss of control (4-point scale from “*Every day*” to “*Less than once per month*”), and distress over binge eating (4-point scale from “*Not at all*” to “*A lot*”). These items were combined to determine a severity score on a 4-point ordinal scale. Higher values are interpreted as “being better,” and lower values as “being worse” about binge eating.

#### 2.4.3. Disordered eating attitudes.

The Disordered Eating Attitude Scale (DEAS) was used, which is a 25-item questionnaire validated for Brazilian women [34] and for men [35] to evaluate one’s relationship with food, involving beliefs, thoughts, feelings, and behaviors.

In the present study, two subscale scores were used. The 4-item subscale “Concerns about food and weight gain” (CFWG) evaluates concerns about calories, intake control, weight gain, and obsessive thoughts about food (e.g., “Do you worry about how much a certain kind of food or meal will make you gain weight?”). The 4-item subscale “Restrictive and compensatory practices” (RCP) measures strict food restriction (e.g., “Have you ever gone one or more days without eating or having only liquids to lose weight?”). Participants rated their agreement using a “Yes or No” scale for binary questions and a Likert scale from 1 (never) to 5 (always) for graduated questions, with item 12 ranging from 1 (Restart eating as usual) to 5 (Use some kind of compensation, such as physical activity, vomiting, laxatives, and diuretics). The sum of item responses gives the scale scores, with higher scores indicating more disordered eating behaviors. In the present sample, the 8-item and 2-factor model with CFA showed excellent fit [CFI = .989, TLI = .984, RMSEA = .049 (90% CI:.024−.072), SRMR = .062, χ^2^_(19)_ = 37.0], with standardized factor loadings ranging from.63 to.91 and a factor correlation of.56; internal consistency reliability was α of.80 and ω of.81 for CFWG and α of.65 and ω of.67 for RCP (mean inter-item correlation = .32).

### 2.5.Self-esteem

We used the Rosenberg Self-Esteem Scale (RSES) [36], which was validated for the Brazilian population [37]. It comprises ten items related to feelings regarding self-esteem and self-acceptance (e.g., “I think I have several good qualities”). Items are rated on a 4-point Likert scale from “*Strongly disagree*” to “*Strongly agree*.” The final score is obtained by summing item responses after reversing the negatively worded items, with higher scores indicating higher self-esteem. The 10-item and 1-factor model with CFA showed satisfactory fit, except for RMSEA [CFI = .954, TLI = .941, RMSEA = .197 (90% CI:.183−.212), SRMR = .072, χ^2^_(39)_ = 578.0], with standardized factor loadings ranging from.66 to.95; internal consistency reliability in the present sample was excellent (Cronbach’s α and McDonald’s ω = .91).

### 3. Scale adaptation of the WBIS-M

The WBIS-M translation followed the 6-step guidelines for test adaptation recommended for use in the Brazilian context [38]. It was also based on recommendations about the pre-condition, test development, and confirmation from the International Test Commission (ITC) guidelines [39]. Initially, the study’s original author authorized the validation study in Brazil. Next, three independent, bilingual speakers forward-translated the WBIS-M from English into Brazilian Portuguese in the first step. The research team synthesized the three forward translations in the second step through a consensual approach. In the third step, a committee of experts in the fields of body image, obesity, and eating behavior, consisting of seven researchers, evaluated all prepared material regarding semantic, idiomatic, cultural, and conceptual equivalence, rating each WBIS-M item on a 3-point scale (1 =* Appropriate,* 0 = *Moderately appropriate,* – 1 = *Inappropriate/Requires modification).* If an item was rated as appropriate by more than five experts, it was rated “A”, and if an item was rated as inappropriate by two experts, it was rated “B”. When two or more committee members rated an item as −1, leading to a “B” rating, the item was revised through a consensual approach by the present study’s three Brazilian authors. In the fourth step, the Brazilian WBIS-M was pre-tested for clarity and comprehension of items, response format, and instructions with ten individuals (four women and six men) who matched the characteristics of the target sample. In two focus groups divided by sex, these participants completed the WBIS-M and discussed the degree of relevance, representativeness, clarity, and comprehensiveness. In their own words, they were asked to describe what they understood from each item and if they experienced any difficulties understanding any item. Based on the discussions, the research team made minor adjustments to the Brazilian Portuguese version of the WBIS-M. In the final step, two independent bilingual speakers back-translated this version of the WBIS-M into English. The two back-translations were then synthesized into a single version and submitted to the original WBIS-M author for final approval.

### 4. Statistical analysis

Analyses were performed with the SPSS24, Mplus8.9, and Stata18.0 programs. First, regarding internal structure, we used a cross-validation design to determine the dimensionality of the 11 initial WBIS-M items. This was done by splitting the sample randomly into two subsamples of approximately the same size; this yielded samples with *N*s slightly above 200, which can be considered above the minimum acceptable size for factor analysis [40]. In the first subsample, exploratory factor analysis (EFA) with the extraction of one and two factors was conducted, the latter with geosmin-rotation, after verifying that a minimum KMO (Kaiser-Meyer-Olkin) of.70 was achieved. Only factors with an eigenvalue higher than one were retained, and Cattell’s scree test and parallel analysis were applied to the number of factors. Acceptable salient loadings were considered above.40. For multidimensional solutions, items showing cross-loading would be allocated to the factor with the highest loading when the difference concerning the second highest value (in absolute value) is above.10; otherwise, the item’s contribution to internal consistency reliability would be considered. In the second subsample, confirmatory factor analysis (CFA) was conducted to test if the EFA’s best solution could be replicated. For both types of analysis, the Weighted Least Squares Means and Variance adjusted (WLSMV) method of estimation for categorical indicators and theta parameterization was applied [41]. Goodness-of-fit was evaluated with χ^2^, CFI (Comparative Fit Index), Tucker-Lewis Index (TLI), Root Mean Square Error of Approximation (RMSEA), and SRMR (Standardized Root Mean Square Residual); cut-off-points used were CFI and TLI > .90 and RMSEA and SRMR < .08 for indication of a satisfactory fit [40]. Second, multigroup CFA was conducted to test measurement invariance across genders at metric (equivalence of factor loadings) and scalar (equivalence of item thresholds) levels [42]. We used the fixed-factor method for model identification as detailed in Ezpeleta and Penelo (2015) [43], and invariance was examined both with the chi-square difference for nested models (difftest option of Mplus, α level set at.01) and the difference in CFI and RMSEA (decrement in CFI > .010 and increment in RMSEA > .015 as indicators of non-invariance) [44].

Next, internal consistency reliability was assessed using McDonald’s omega and Cronbach’s alpha coefficients. Lastly, relationships between the WBIS-M scores and external measures were analyzed using *Pearson correlations* for quantitative measures, *Spearman correlations* for ordinal variables, and t-test for comparing groups (binary independent variable and quantitative dependent variable) and Cohen’s *d* to value the effect size of the difference between group means.

## Results

### 1. Cross-cultural adaptation

The instrument’s cross-cultural adaptation from English to Brazilian Portuguese did not present significant issues or difficulties in understanding. Table 2 describes the classification according to the expert committee’s semantic, idiomatic, cultural, and conceptual equivalence. No item was rated as inappropriate by more than two experts. The most challenging questions in the first phase were the scale title and question 2. One expert suggested changing “weight stigma” to “body weight-related stigma” in the title, but the scale title was kept as it originally was due to the growing number of studies using the term. Regarding question 2, the suggestion was to remove the word “others” to make more sense.

**Table 2:** Classification according to semantic, idiomatic, cultural, and conceptual equivalence.

Original/English Version	Brazilian Version	Classification
Title: Modified Weight Bias Internalization Scale	*Título: Escala de internalização do estigma do peso modificada*	B
1. Because of my weight, I feel that I am just as competent as anyone *	1. Por causa do meu peso, eu sinto que sou tão competente quanto qualquer outra pessoa *	A
2. I am less attractive than most other people because of my weight	*2. Eu sou menos atraente do que a maioria das pessoas por causa do meu peso*	B
3. I feel anxious about my weight because of what people might think of me	*3. Eu me sinto ansioso(a) sobre o meu peso por causa do que as pessoas podem pensar de mim*	A
4. I wish I could drastically change my weight	*4. Eu gostaria de poder mudar drasticamente meu peso*	A
5. Whenever I think a lot about my weight, I feel depressed	*5. Sempre que eu penso muito sobre meu peso, me sinto deprimido(a)*	A
6. I hate myself for my weight	*6. Eu me odeio por causa do meu peso*	A
7. My weight is a major way that I judge my value as a person	*7. Meu peso é a principal forma pela qual eu julgo meu valor como pessoa*	A
8. I don’t feel that I deserve to have a really fulfilling social life because of my weight	*8. Eu não sinto que mereço ter uma vida social plena, por causa do meu peso*	A
9. I am OK being the weight that I am *	9. Eu estou bem com o peso que tenho *	A
10. Because of my weight, I don’t feel like my true self	*10. Por causa do meu peso eu não me sinto como eu sou de verdade*	A
11. Because of my weight, I don’t understand how anyone attractive would want to date me	*11. Por causa do meu peso, eu não entendo como alguém atraente poderia querer namorar comigo*	A

* Reversed item.

Note. Experts: *n* = 7; A: Zero or one expert rated the equivalence of the item as inadequate; B: Two experts rated the equivalence as inadequate.

In the focus group, the problematic items were 1, 3, and 10 due to the words “competent,” “anxious,” and “my true self,” respectively. The participants questioned the possibility of different interpretations for each person. The items were originally maintained as the instrument requires the respondent’s internal self-interpretation. The volunteers also asked about the instrument’s many response options, but they valued the different intensities.

### 2. Descriptive analyses of items

Missing responses for the 11 WBIS-M items were very low (0.46%), with only 17 participants (4.1%) exhibiting missing values for one or more items, which supported the use of the entire information method for factor analysis, the item mean substitution method for rounding off to discrete values at the scale level, and later the list-wise deletion method for correlation analysis with the other test scores [45]. Mean values (and *SD*) for WBIS-M item responses ranged from 1.75 (1.37) to 3.88 (1.99), whereas items 7 and 8 showed greater skewness (> 2) and kurtosis (> 4) in absolute value than the rest of the items (Table 3, left).

**Table 3:** Descriptive statistics of items in the whole sample, factor loadings with EFA and CFA in each subsample, and parameter estimates after measurement invariance analysis across gender.

Abbreviated items	Descriptives (*N* = 418)				Standardized factor loadings (subsamples)		Invariance: Factor loadings (across gender)	
	N	M (SD)	Skewness	Kurtosis	EFA (n = 206)	CFA (n = 212)	Unstandardized	Standardized
1. Feel competent*	416	3.85 (2.18)	0.15	−1.36	.006	–	–	–
2. Less attractive	417	3.36 (2.06)	0.26	−1.40	.870	.854	1.857	.880/.845
3. Anxious people think	416	3.58 (2.18)	0.17	−1.49	.828	.848	1.591	.847/.804
4. Wish change	417	3.66 (2.18)	0.18	−1.45	.861	.876	1.831	.878/.841
5. Depressed I think a lot	415	3.26 (2.14)	0.47	−1.26	.906	.912	2.298	.917/.890
6. Hate myself	415	2.04 (1.66)	1.65	1.67	.918	.916	2.310	.918/.891
7. Judge person value	416	1.80 (1.43)	2.18	4.16	.938	.885	1.695	.861/.821
8. Don’t deserve fulfilling social life	414	1.75 (1.37)	2.14	4.00	.834	.876	1.767	.870/.832
9. Being OK*	417	3.88 (1.99)	0.06	−1.37	.818	.773	1.360	.806/.756
10. Don’t feel true self	416	2.47 (1.83)	1.02	−0.22	.838	.866	1.716	.864/.825
11. Don’t understand dating me	418	2.71 (2.05)	0.84	−0.74	.884	.884	1.995	.894/.861

* Reversed item.

Note. EFA: Exploratory Factor Analysis; CFA: Confirmatory Factor Analysis.

### 3. Internal structure and internal consistency reliability

Regarding the cross-validation design for factor analyses, no differences were observed for any of the demographic characteristics between the two random sub-samples (*p* > .213). For the first sub-sample (*n* = 206), Bartlett’s test of sphericity showed that data were suitable for EFA (KMO = .918; χ^2^_(55)_ = 1594.8, *p *< .005). The first two observed eigenvalues were above 1 (7.493 and 1.039). From the third, the eigenvalues were below 1 (≤ 0.687). If MLR were used, only the first observed eigenvalue would be larger than the average generated from parallel analysis (option not available in Mplus for categorical indicators). Cattell’s scree test also suggested the extraction of only one factor. The 11-item and 1-factor model with EFA showed a nearly acceptable fit, except for RMSEA [CFI = .981, TLI = .977, RMSEA = .121 (90% CI:.103−.140), SRMR = .055, χ^2^_(44)_ = 176.5]; all factor loadings were above.40 (.82−.92) and statistically significant (*p* < .001), except for item 1 (.01, *p* > .05). Although the 11-item and 2-factor model with EFA showed better fit [CFI = .994, TLI = .990, RMSEA = .081 (90% CI:.058−.104), SRMR = .032, χ^2^_(34)_ = 79.4], it also showed a high factor correlation (which could indicate some overlap between factors), item 1 continued without loading saliently on either factor (≤.10 in absolute value, *p* > .05), several items showed cross-loadings (mainly items 6 and 8, and to a lesser extent item 10), and the pattern of factor loadings was not interpretable. Therefore, in the second sub-sample (*n* = 212), CFA was conducted to test a 10-item and 1-factor model, after removing item 1. This model showed a satisfactory fit, except again for RMSEA [CFI = .990, TLI = .988, RMSEA = .096 (90% CI:.075−.117), SRMR = .025, χ^2^_(35)_ = 103.1] and all standardized factor loadings were high (.77−.92) and statistically significant (*p* < .001) (Table 3, center).

Next, we evaluated the measurement invariance of the selected 10-item and 1-factor model across gender (253 women and 160 men, not considering the 3 participants who responded to the category “other”). Full metric [∆Χ^2^_(9)_ = 17.1, *p *= .047; ∆CFI = .004; ∆RMSEA = −0.023] and full scalar invariance [∆Χ^2^_(59)_ = 86.2, *p *= .012; ∆CFI = −.001; ∆RMSEA = −0.020] were achieved, since all factor loadings (Table 3, right) and all item thresholds (details upon request), respectively, were equivalent across gender. Hence, meaningful comparisons of scores among women and men can be conducted [42].

Internal consistency reliability for the final 10-item WBIS-M version was excellent, both for the whole sample and the subsamples, with a value of.94 both for McDonald’s omega and Cronbach’s alpha. Moreover, item analysis showed that, after removal of item 1, all the items contributed to internal consistency (item-total corrected correlation ≥ .67), and none decreased the reliability coefficient, whereas maintenance of item 1 would decrease omega and alpha values (both from.94 to.92).

### 4. Relationships with external variables

Table 4 shows descriptive statistics for the WBIS-M total score, obtained by averaging items 2–11, and correlation coefficients with external measures. As expected, regarding convergent and discriminant validity, WBIS-M scores were highly and positively related to BSQ-8-Body dissatisfaction and negatively related to RSES-Self-esteem scores (*r* = .60−.86, in absolute value), positively and moderately related to DEAS-Restrictive and compensatory behaviors (*r* = .47) and negatively and moderately related to less severe Binge eating (*r *= −.41), positively and weakly related to DEAS-Concern with food and weight gain scores (*r* = .36), and almost unrelated to AFAT-Social and character depreciation scores (*r* = .11). In addition, the association with BMI was moderate (*r* = .44). There was no relationship with age (*r *= −.01).

**Table 4:** Descriptive statistics and correlations of WBIS-M scores and quantitative external measures.

Measure	(min/max)	*M* (*SD*)	WBIS-M correlation	
			*r*	*r*_c_
WBIS-M Total score	(1–7)	2.85 (1.54)	–	–
AFAT Social and character depreciation subscale	(1–5)	1.30 (0.37)	.09	.11
DEAS Concern with food and weight gain subscale	(4–20)	7.66 (3.65)	.31*	.36
DEAS Restrictive and compensatory practices subscale	(4–20)	6.08 (3.61)	.47*	.60
Binge Eating			−.41**^a^	
RSES self-esteem total score	(10–40)	31.30 (6.43)	−.63*	−.68
BSQ-8 body shape questionnaire total score	(1–6)	2.86 (1.18)	.79*	.86
Body Mass Index (kg/m²)	(15–60)	25.86 (5.45)	.44*	–
Age		30.8 (10.4)	−.01	–

*r*_c_: correlation corrected for attenuation due to unreliability; * *p* < .05; ^a^ Spearman correlation because Binge Eating is an ordinal variable.

Note: WBIS-M: Modified Weight Bias Internalization Scale; AFAT: Anti-fat Attitudes Scale; DEAS: Disordered Eating Attitudes Scale; RSES: Rosenberg Self-Esteem Scale; BSQ-8: Body Shape Questionnaire Short Version.

Regarding gender, the category “other” was not included in the mean comparison test because of its very low size in the study sample (*n* = 3). Women (*M *= 3.08, *SD* = 1.62) scored significantly higher than men [*M* = 2.48, *SD* = 1.31; *t* (384.9) = 4.14; *p* < .001], despite the effect size being small (*d* = 0.40).

## Discussion

The main objectives of this study were fulfilled, and the instrument presented a successful adaptation from English to Brazilian Portuguese without many doubts and questions from the experts and the focus group. Moreover, the Brazilian Portuguese translation of the WBIS-M presented a good factor structure for the 10-item and 1-factor model, with good internal consistency reliability for the total score, as expected. The current factor structure differs from the ones found in previous studies [13,18–20,45]. However, it is consistent with the results of Andrés et al. [11] and Lee and Dedrick [22].

In our EFA, factor loadings ranged from.00 to.87 due to the reverse item 1 (“Because of my weight, I feel that I am just as competent as anyone”) having a low and non-significant factor loading. Therefore, we excluded this item for CFA, and this 10-item and 1-factor model showed factor loadings ranging between.70−.85. This exclusion is consistent with previous validation studies in different samples, such as adolescents [11] and adults [22,46]. The excluded item is a reverse question and can be problematic when translating from English to other languages as it can cause confusion and ambiguity [47,48]. Measurement invariance analysis across genders showed that WBIS-M works in the same way for women and men. Our results also provide satisfactory evidence in terms of internal consistency reliability (α = .94 and ω = .94) and adequate convergent and discriminant validity since WBIS-M scores were significantly associated with higher body dissatisfaction, lower self-esteem, and worse disordered eating behavior, as expected.

The association between WBIS-M scores and anti-fat attitudes was almost zero, indicating that internalized weight stigma is not associated with public stigma. This result aligns with conceptual differences between the interpersonal and intrapersonal components [4]. Based on previous studies showing a low correlation between internalized stigma and disliking PLWO [10–12], we hypothesized that the correlation would be significant but weak.

Internalized weight stigma was strongly associated with body dissatisfaction and moderately inversely with self-esteem. It leads to mental health issues, reduced self-care, greater body dissatisfaction, and lower self-esteem [7,8,49]. These results mirror previous studies [10–12], although Durso et al. [46] found a moderate correlation with body dissatisfaction and a weak association with self-esteem. Internalized stigma and thin ideals can increase body dissatisfaction [5,7,46].

Regarding disordered eating, greater internalization of weight stigma correlates with more severe binge eating, restrictive and compensatory behaviors, and greater concerns about food and weight gain. The weak correlation with concerns about food and weight gain aligns with studies showing moderate or slight association with dieting, pursuit of thinness, and binge eating [10–12]. Belief in thin ideals can lead to more restrictive diet practices [5,7], and the internalization of weight stigma contributes to disordered eating behaviors like binge eating [13], restrictive eating [15], and weight loss practices [7].

According to our hypothesis, women showed greater internalization of weight stigma. Differences in stigma according to gender were previously described, reaffirming that women present greater internalization and weight stigma [18,25,26,50]. Greater internalization may occur in people with greater beliefs about thin ideals, as it occurs in a greater proportion of women [5,7].

This study showed that the association between WBIS-M and BMI was moderate since BMI is described as a predictor of internalized stigma in the study realized by Puhl et al. [13], which explains that a higher BMI predicts greater internalized stigma. Previous studies have shown a similar result regarding the strength and direction of the correlation between both measures [5,7,12]. However, two studies did not find a significant result for this potential association in an adult population [10,46], and Andrés et al. [11] found a weak association with BMI in a study with adolescents, but this can also be attributed to the fact that BMI variability is different in this sample.

A recent study in adolescents demonstrated a difference between underweight and overweight status in relation to the normal weight status, according to the BMI classification, showing that these groups have a higher internalization of weight stigma [50]. In adults, a study found that the obesity status was the group with the highest self-stigma, reinforcing that the highest risks of greater internalization of weight bias are at higher BMIs [14].

Different from what was expected, no relationship with age was observed, contrary to the hypothesis that the older the age, the less the internalized stigma. However, the lack of association may be due to sampling bias, such as recruiting individuals with similar levels of interest in weight-related issues and although the age range is 18–68, there is no excessive variability in age in our sample, which comprises a larger portion of young adults, and this portion of the sample may not have been sufficiently different to find associations of greater internalized stigma with age.

As in the present study, another study also found no association of internalized weight stigma with age [5], and a study consisted of adults who were overweight and enrolled in a behavioral weight loss program [46]. Nevertheless, some studies show an association of greater internalized stigma in younger people [7,13], younger people who are overweight [7], and older adults over 65 years old who have less self-stigma [14]. It is necessary to understand better this relationship between internalization and age groups regarding thin ideals, body concerns, and stigmatizing experiences.

This study has limitations, including the self-reporting nature of online data and potential response desirability bias, despite anonymity. The sample was not representative of all Brazilian adults; it mainly included women, White people, and postgraduates. In addition, having split the sample into two sub-samples for EFA and CFA ensured independence between exploratory and confirmatory steps. However, we acknowledge that having recombined the full sample for measurement invariance testing across genders may have introduced overfitting and/or inflated model fit indices, as the factor structure was already determined from part of the same data. No studies on measurement invariance across genders were found for the Brazilian versions of other instruments utilized. Therefore, we recommend further studies that can analyze differences in sexual orientation, ethnicity, income, age group, and educational level with representative samples, as well as continuing to study the associations of internalized weight stigma with physical and mental health issues in different populations and contexts.

The study’s strengths include being the first to validate an instrument for assessing internalized weight stigma in the Brazilian population, including different weight statuses. It contributes to understanding stigma across cultures, particularly in low- and middle-income countries.

This study supports that the Brazilian Portuguese adaptation meets expectations for language adaptation and content validity. It presents a unidimensional structure with a good fit, replicating the original 1-factor model. Thus, the 10-item WBIS-M is a psychometrically valid and reliable measure for adult Brazilian samples. Test scores behaved as expected concerning relations between stigma internalization and external variables. Thus, internalized weight stigma was strongly associated with body dissatisfaction, moderately with self-esteem, and weakly to moderately with disordered eating. Finally, women and people with higher BMI had greater internalized weight bias, whereas the association with age was negligible.

This study identified key directions for future research and findings highlight the relevance of assessing internalized weight stigma in both research and clinical settings in Brazil. Given its well-documented impact on physical, psychosocial, and behavioral health, having a validated instrument for the Brazilian population is essential. The Brazilian WBIS-M is suitable for use in longitudinal research and diverse Brazilian populations, allowing for a deeper understanding of stigma’s long-term effects. Clinically, this scale can serve as a screening measure to identify individuals experiencing weight-related self-devaluation. Additionally, data from the WBIS-M could contribute to the evidence base for developing targeted interventions in policies and programs in education, regulation, and legislation, with particular focus on women, individuals with obesity, and those facing challenges related to body image and eating behaviors. These findings reinforce the importance of developing inclusive approaches to weight-related discussions and intervention strategies that reduce self-stigma, ultimately mitigating its harmful health consequences and improving the well-being of affected individuals, as recommended in the recent international consensus statement for ending obesity stigma [1] and in the World Obesity Federation’s statement, which highlighted the need for strategies in low- and middle-income countries [23].

## Supporting information

S1 File Brazilian version of the Weight Bias Internalization Scale (WBIS-M). Versão brasileira da Weight Bias Internalization Scale (WBIS-M), Escala de Internalização do Estigma do Peso Modificada.
(DOCX)
