# Supplementary material for: Adaptation and validation of the modified weight bias internalization scale (WBIS-M) in Brazilian adults
Source: PLoS One. 2025 Jul 31;20(7):e0328176. doi: 10.1371/journal.pone.0328176 (PMC12312967; doi:10.1371/journal.pone.0328176)
Supplement: S1 File — Versão brasileira da Weight Bias Internalization Scale (WBIS-M), Escala de Internalização do Estigma do Peso Modificada. (DOCX) [file pone.0328176.s001.docx]

**S1 File. Brazilian Version of the Weight Bias Internalization Scale (WBIS-M)**

Versão brasileira da *Weight Bias Internalization Scale* (WBIS-M), Escala de Internalização do Estigma do Peso Modificada

Instruções: Por favor, avalie seu nível de concordância com as seguintes afirmações usando esta escala:

1 = Discordo fortemente
2 = Discordo
3 = Discordo um pouco
4 = Nem concordo nem discordo
5 = Concordo um pouco
6 = Concordo
7 = Concordo fortemente

| Por favor, avalie seu nível de concordância com as seguintes afirmações usando esta escala: | Discordo  fortemente | | | Concordo  fortemente | | | |
| --- | --- | --- | --- | --- | --- | --- | --- |
|  | 1 | 2 | 3 | 4 | 5 | 6 | 7 |
| 2.Eu sou menos atraente do que a maioria das pessoas por causa do meu peso. |  |  |  |  |  |  |  |
| 3.Eu me sinto ansioso (a) sobre o meu peso por causa do que as pessoas podem pensar de mim. |  |  |  |  |  |  |  |
| 4.Eu gostaria de poder mudar drasticamente meu peso. |  |  |  |  |  |  |  |
| 5.Sempre que eu penso muito sobre meu peso, me sinto deprimido (a). |  |  |  |  |  |  |  |
| 6.Eu me odeio por causa do meu peso. |  |  |  |  |  |  |  |
| 7.Meu peso é a principal forma pela qual eu julgo meu valor como pessoa. |  |  |  |  |  |  |  |
| 8.Eu não sinto que mereço ter uma vida social plena, por causa do meu peso. |  |  |  |  |  |  |  |
| 9.Eu estou bem com o peso que tenho.^1^ |  |  |  |  |  |  |  |
| 10.Por causa do meu peso eu não me sinto como eu sou de verdade. |  |  |  |  |  |  |  |
| 11.Por causa do meu peso, eu não entendo como alguém atraente poderia querer namorar comigo. |  |  |  |  |  |  |  |

^1^ Item inverso

O item 1 original foi eliminado devido a um funcionamento psicométrico atípico.

Manteve-se a numeração original, com os itens de 2 a 11, para preservar a coerência com os nomes das variáveis no banco de dados.

Os itens são avaliados em uma escala Likert de 7 pontos, onde 1 = Discordo totalmente; 7 = Concordo totalmente.

A pontuação total, obtida pela soma dos 10 itens (após a recodificação do item 9, que é inverso), é dividida por 10, de forma que a pontuação final varia entre 1 e 7, sendo 4 a média teórica. Pontuações mais altas indicam maior grau em que as pessoas aplicam estereótipos baseados no peso a si mesmas e baseiam suas autoavaliações no peso.

Instrumento original:

Pearl, R. L., & Puhl, R. M. (2014). Measuring internalized weight attitudes across body weight categories: Validation of the Modified Weight Bias Internalization Scale. *Body Image, 11*(1), 89–92. <https://doi.org/10.1016/j.bodyim.2013.09.005>
